# The Efficacy and Safety of Monoclonal Antibodies That Target Alpha‐Synuclein in Parkinson’s Disease: A Systematic Review

**DOI:** 10.1155/padi/8540084

**Published:** 2026-07-22

**Authors:** Daniel Coles, Anish Kalyana, Sameer Khalil, Dhyana Chauhan, Thiara Rupasinghe, Charlie Costello, Amit Batla, Tim Young

**Affiliations:** ^1^ School of Medicine, University College London (UCL), London, UK, ucl.ac.uk; ^2^ Royal Free London NHS Foundation Trust, London NW3 2QG, UK, nhs.uk; ^3^ Cambridge Institute for Medical Research, University of Cambridge, Keith Peters Building Cambridge Biomedical Campus, Cambridge, UK, cam.ac.uk; ^4^ Department of Clinical and Movement Neuroscience UCL Institute of Neurology, Queen Square, London WC1N 3BG, UK; ^5^ Queen Square Institute of Neurology UCL, London, UK

**Keywords:** disease-modifying therapy, immunotherapy, monoclonal antibodies, Parkinson’s disease, systematic review

## Abstract

**Background:**

Parkinson’s disease is a progressive neurodegenerative disorder with no currently approved disease‐modifying therapies. Alpha‐synuclein targeting monoclonal antibodies provide a potential therapeutic strategy.

**Objectives:**

To evaluate published studies of the efficacy and/or safety of monoclonal antibodies that target alpha‐synuclein in human subjects.

**Methods:**

A systematic review of peer‐reviewed journal articles was conducted. PubMed, Embase and Scopus were searched up to March 2, 2025. Results were synthesised narratively. Risk of bias was assessed, and sensitivity analysis excluding studies with high risk was performed.

**Results:**

After screening 1509 papers, 10 publications incorporating a total of 13 studies were included. These assessed Prasinezumab, Cinpanemab, Exidavnemab and Lu‐AF82422 with heterogeneity amongst studies. Tolerability was generally favourable across all studies. Cinpanemab showed almost no efficacy, whilst Prasinezumab demonstrated mixed motor symptom improvements. Safety profiles for all monoclonal antibodies reflected mostly consistent rates of adverse events. Six studies were removed in the sensitivity analysis due to high risks of bias, which reduced Prasinezumab’s apparent efficacy findings.

**Conclusions:**

The efficacy of monoclonal antibodies in Parkinson’s disease remains uncertain with most positive results coming from the studies with high risks of bias. Prasinezumab demonstrated an efficacy profile with the potential of significance, warranting further research. This lack of efficacy reported with Cinpanemab is consistent with the manufacturer’s decision to discontinue it. Safety data on Exidavnemab and Lu‐AF82422 in healthy volunteers support further investigation in Parkinson’s disease patients. Future trials may benefit from the inclusion of subjects at earlier disease stages, diagnosed before clinical features have emerged.

**Trial Registration:** ClinicalTrials.gov identifier: NCT03100149

## 1. Introduction

Parkinson’s disease (PD) is the second most common neurodegenerative condition worldwide, yet currently only symptomatic rather than disease‐modifying treatment is available. [1, 2].

PD patients commonly experience symptoms such as tremors, rigidity and bradykinesia, sleep disturbances and olfactory dysfunction [3, 4]. Current understandings of pathogenesis are that alpha‐synuclein misfolds and aggregates, forming Lewy bodies [5]. These contribute to neurodegeneration, particularly in the substantia nigra, reducing dopamine production [6]. This is thought to be the main driving force behind many symptoms, e.g., tremor [6].

Current PD treatments are symptomatic [2]. Levodopa remains the gold standard, improving motor function by increasing striatal dopamine [2, 7]. However, disease progression and desensitisation often necessitate higher doses, increasing the risk of serious adverse effects (AEs) [2]. This limitation underscores the need for disease‐modifying therapies (DMTs).

DMTs for PD are not yet approved for routine use but are under investigation [8]. Alpha‐synuclein–targeting mAbs are a current avenue being researched [9]. They have already demonstrated significant efficacy in cancers, autoimmune disorders and infections with generally favourable safety profiles. [10].

This study aims to provide an up‐to‐date systematic review of clinical trials and secondary analyses on the efficacy and/or safety of alpha‐synuclein–targeting mAbs in PD patients and healthy volunteers (HVs).

## 2. Methods

This systematic review was conducted in accordance with the Preferred Reporting Items for Systematic Reviews and Meta‐Analyses (PRISMA) guidelines. [11].

### 2.1. Literature Search Strategy

**FIGURE 1:**
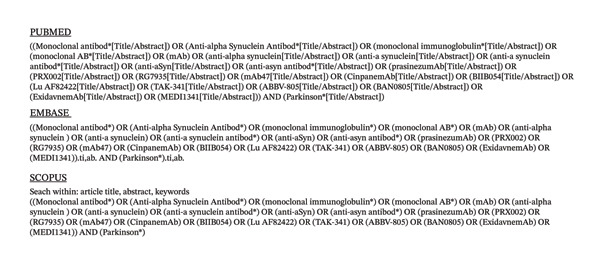
Search terms. Abbreviations: Ab, antibody; asyn, alpha‐synuclein; a‐synuclein, alpha‐synuclein; mAb, monoclonal antibody.

Specific search terms (Figure 1) were applied to titles and abstracts in PubMed, Embase and Scopus up until March 2nd, 2025. The articles were imported into Rayyan for review.

### 2.2. Inclusion and Exclusion Criteria

**FIGURE 2:**
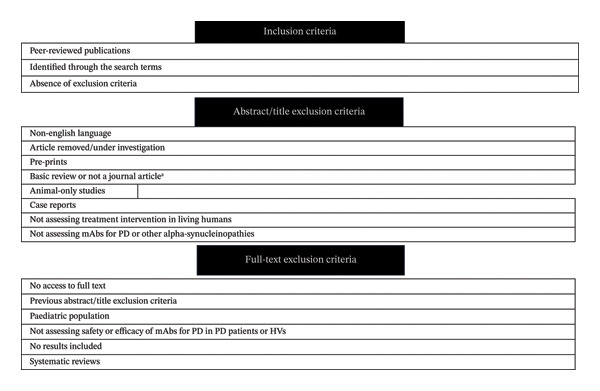
Inclusion and exclusion criteria for study selection. ^a^Exclude any nonsystematic reviews and nonarticle formats, e.g., editorials or short surveys. Abbreviations: HVs, healthy volunteers; mAb, monoclonal antibody; PD, Parkinson’s disease.

**FIGURE 3:**
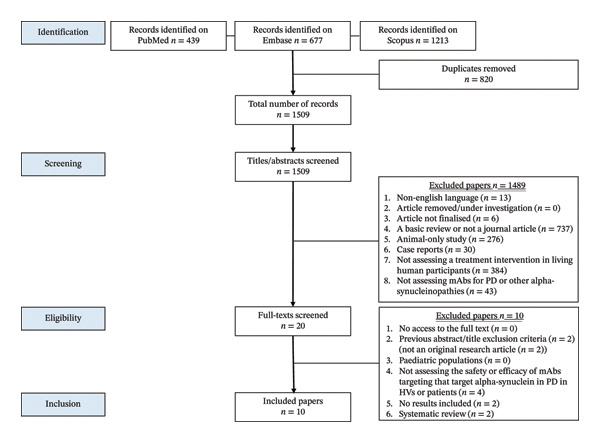
PRISMA flow diagram [11]. Abbreviations: HVs, healthy volunteers; mAb, monoclonal antibody; PD, Parkinson’s disease.

Inclusion and exclusion criteria are summarised in Figure 2. Exclusion criteria were applied sequentially, independently by five reviewers, and final inclusion of each article was decided by consensus where required. Excluded articles were labelled according to the highest‐level of exclusion criteria. Relevant systematic reviews or studies on other alpha‐synucleinopathies were retained for full‐text screening. The screening results are included in a PRISMA flow diagram (Figure 3) [11].

### 2.3. Data Extraction

The findings from distinct trials or extensions were analysed separately. Papers with overlapping cohorts were marked with an asterisk. No meta‐analysis was performed due to study heterogenicity.

Trial methodologies and outcomes are summarised in Table 1, including primary and secondary outcomes related to efficacy or safety. Primary efficacy outcomes were reported with confidence intervals, and statistically significant secondary outcomes include accompanying data; insignificant findings were grouped. Safety was primarily assessed via treatment‐related adverse events (TRAEs), or AEs if TRAEs were unavailable. Notable trends were highlighted; remaining findings labelled “nothing clinically relevant”.

**TABLE 1:** Study findings.

Reference, monoclonal antibody and trial name if applicable	Study design	Length of observation	Populations breakdowns and comparators (*n* = number of participants)	Participants not included in final analysis (*n* = number of participants)	Relevant outcomes	Primary outcomes	Secondary outcomes
Schenk et al., 2017 Prasinezumab	RCT ‐ Randomised ‐ Double‐blind ‐ Single‐centre	16 weeks	Total: *n* = 40 HVs Intervention (*n* = 30) 0.3 mg/kg (*n* = 6) 1 mg/kg (*n* = 6) 3 mg/kg (*n* = 6) 10 mg/kg (*n* = 6) 30 mg/kg (*n* = 6) Comparator: Placebo (*n* = 10)	At week 12: *n* = 1 (3.33%) • 1 in 0.3 mg/kg (3.33%)	Primary: safety assessed through TRAEs, AEs, severe AEs, death, laboratory tests, vital signs, ECG and physical examination secondary: NR	TRAEs: *n* = 5 (12.5%) 1 in placebo (10.0%), 4 in intervention (13.3%) By intervention group: 1 in 1 mg/kg (16.7%), 3 in 10 mg/kg (50.0%) AEs: *n* = 13 (43.3%), severe AEs: *n* = 1 (deemed unrelated), deaths: *n* = 0 laboratory tests, vital signs, examination, ECG: nothing clinically relevant	NR

Jankovic et al., 2018 Prasinezumab	RCT ‐ Randomised ‐ Double‐blind ‐ Multicentre	24 weeks	Total: *n* = 80 PD patients Intervention (*n* = 55) 0.3 mg/kg (*n* = 8) 1 mg/kg (*n* = 8) 3 mg/kg (*n* = 8) 10 mg/kg (*n* = 8) 30 mg/kg (*n* = 11) 60 mg/kg (*n* = 12) Comparator: Placebo (*n* = 25)	At week 24: *n* = 8 (10.0%) • 1 in 0.3 mg/kg (12.5%) • 1 in 30 mg/kg (9.09%) • 3 in 60 mg/kg (25.0%) • 3 in placebo (12.0%)	Primary: Safety assessed through TRAEs, AEs, severe AEs, death, laboratory tests, vital signs, ECG and physical and neurological examinations secondary: NR	TRAEs: *n* = 7 (12.7%) 3 in placebo (12.0%), 6 in intervention (10.9%) By intervention group: 1 in 0.3 mg/kg (12.5%), 1 in 3 mg/kg (12.5%), 4 in 60 mg/kg (33.3%) AEs: *n* = 37 (67.3%), severe AEs: *n* = 0, deaths: *n* = 0 laboratory tests, vital signs, examinations, ECG: nothing clinically relevant	NR

Pagano et al., 2022 Prasinezumab PASADENA Trial‐ Part 1^∗^	RCT ‐ Randomised ‐ Double‐blind ‐ Multicentre	52 weeks	Total: *n* = 316 PD Patients Intervention (*n* = 211) Low‐dose: 1500mg (*n* = 105) High‐dose: 4500 mg when ≥ 65 kg or 3500 mg when < 65 kg (*n* = 106) Comparator: Placebo (*n* = 105)	At week 52: *n* = 96 (30.4%) • 32 in low‐dose (30.5%) • 33 in high‐dose (31.1%) • 31 in placebo (29.5%)	Primary: Efficacy Assessed through change in MDS‐UPDRS Combined Score (Parts 1, 2 & 3) Secondary: Efficacy and Safety Efficacy assessed using MDS‐UPDRS Parts 1, 2 and 3 separately, DaT‐SPECT, MoCA, CGI‐1, PGI‐C, SE‐ADL, Time to start of dopaminergic treatment, and time to first occurrence of ≥ 3‐point increase from baseline in MDS‐UPDRS Part s1 or 2. Safety assessed through AEs, severe AEs, ECG, MRI abnormalities, blood pressure and laboratory tests.	MDS‐UPDRS combined score: Low‐dose vs placebo adjusted mean difference (80% CI) −2.00 (−4.20–0.20) High‐dose vs placebo adjusted mean difference (80% CI) −0.60 (−2.80–1.60)	SAFETY TRAEs: *n* = 95 (30.1%) 28 in placebo (26.7%), 26 in low‐dose (24.8%), 41 in high‐dose (38.7%) AEs were ∼10% higher in both treatment groups vs placebo. Serious AEs were low and similar across cohorts; 4 were deemed treatment‐related. No deaths reported. Other safety‐related results were not reported. EFFICACY Most secondary efficacy‐related outcomes reported no significant differences except: MDS‐UPDRS Part 3: Low‐dose vs. placebo mean difference in (80% CI) −1.90 (−3.50–−0.30) MoCA: High‐dose vs. placebo mean difference (80% CI) 0.44 (0.13 to 0.75)

Pagano et al., 2022 Prasinezumab PASADENA Trial‐ Part 2^∗^	Open‐label extension of the PASADENA trial ‐ Blinded to dose allocation ‐ Placebo group becomes delayed‐start cohort, with randomised doses in the same ratio and intervention becomes early‐start cohort ‐ Multicentre	52 week extension (104 weeks total duration)	Total: *n* = 309 PD Patients Delayed‐start cohort (*n* = 94) Low‐dose: 1500 mg (*n* = 52) High‐dose: 4500 mg ≥ 65 kg or 3500 mg < 65 kg (*n* = 53) Early‐start cohort (*n* = 177) Low‐dose: 1500 mg (*n* = 100) High‐dose: 4500 mg ≥ 65 kg or 3500 mg < 65 kg(*n* = 106) Comparators: Early‐ vs. delayed‐start cohort and low‐ vs high‐dose cohort	At week 104 of the total duration: *n* = 223 (72.2%) Early‐start • 74 in low‐dose (74.0%) • 71 in high‐dose (71.0%) Delayed‐start • 38 in low‐dose (73.1%) • 40 in high‐dose (75.5%)	Primary: Efficacy Assessed through change in MDS‐UPDRS Combined Score (Parts 1, 2 & 3) Secondary: Efficacy and Safety Efficacy assessed using MDS‐UPDRS Part 1, 2 and 3 separately, DaT‐SPECT, MoCA, CGI‐1, PGI‐C, SE‐ADL, time to start of dopaminergic treatment, and time to first occurrence of ≥ 3‐point increase from baseline in MDS‐UPDRS Parts 1 or 2. Safety assessed through AEs, Severe AEs, ECG, MRI abnormalities, blood pressure and laboratory tests.	MDS‐UPDRS combined score: Early‐ vs delayed‐start cohort adjusted mean difference (80% CI) −1.78 (−4.66–1.10)	SAFETY TRAEs: *n* = 54 (17.5%) 7 in low‐dose early‐start cohort (7.0%), 9 in low dose delayed‐start cohort (17.3%). 22 in high dose early‐start cohort (21.2%), 16 in high‐dose delayed‐start cohort (30.2%). AEs were similar between early‐ and delayed‐start cohorts. High‐dose groups showed about a 10% higher incidence of AEs. Serious AEs were low and comparable across cohorts; 2 were deemed treatment‐related. 1 death occurred (suicide) but was deemed unrelated. Other safety‐related results were not reported. EFFICACY All secondary efficacy‐related outcomes reported no significant differences.

Pagano, Monnet et al., 2024 Prasinezumab PASADENA Trial^∗^	A comparinson between the PASADENA 3‐year open‐label extension and PPMI observational cohort ‐ Blinded to dose allocation ‐ Early‐start has received Prasinezumab since the RCT began (total exposure: 4 years); delayed‐start began treatment after the RCT ended (total exposure: 3 years) but was still used at the year 1 comparison prior to any intervention‐Multicentre	3 year extension of the 1 year RCT (4 years total duration)	Total: *n* = 271 PD Patients Delayed‐start cohort (*n* = 94) Doses not separately reported Early‐start cohort (*n* = 177) Doses not separately reported Comparator: PPMI observational cohort (*n* = 303)	At year 4 of the total duration:^a^ PD Patients *n* ≈ 29 (10.7%) • ≈10 in Delayed‐start (10.3%) • ≈19 in Early‐start (10.7%) PPMI observational cohort *n* ≈ 183 (60.4%)	Primary: Efficacy Assessed through change in MDS‐UPDRS Parts 2 & 3 Secondary: Efficacy assessed through LEDD, MDS‐UPDRS Part 1 sleep related subscores, MDS‐UPDRS Part 4 and DaT‐SPECT	MDS‐UPDRS Part 3: OFF‐state delayed‐start vs PPMI cohort mean difference (80% CI) −5.73 (−7.33–−4.14), −51% relative difference OFF‐state early‐start vs PPMI cohort mean difference (80% CI) −7.26 (−8.59–−5.93), −65% relative difference ON‐state delayed‐start vs PPMI cohort mean difference (80% CI) −3.71 (−5.41–−2.01), −94% relative difference ON‐state early start vs PPMI cohort mean difference (80% CI) −4.69 (−6.09–−3.30), −118% relative difference MDS‐UPDRS Part 2: Delayed‐start vs. PPMI cohort mean difference (80% CI) −2.20 (−2.96–−1.45), −48% relative difference Early‐start vs. PPMI cohort mean difference (80% CI) −1.82 (−2.44–−1.20), −40% relative difference	Most secondary efficacy‐related outcomes reported no significant differences except: MDS‐UPDRS Part 1: Early‐start vs PPMI cohort mean difference (80% CI) −0.24 (−0.39–−0.09), −47% relative difference Delayed‐start vs PPMI cohort mean difference (80% CI) −0.31 (−0.43–−0.19), −61% relative difference LEDD: Early‐start vs. PPMI cohort mean difference (80% CI) −120.83 mg (−187.68–−53.99) Delayed‐start vs. PPMI cohort mean difference (80% CI) −85.08 mg (−140.01–−30.16)

Pagano, Taylor et al., 2024 Prasinezumab PASADENA Trial^∗^	Post‐hoc analysis of part 1 of the PASADENA trial ‐ Assessing subpopulations that consist of at least 20% of the total population (4 prespecified, 6 exploratory)	52 weeks	Total: *n* = 316 PD Patients Prespecified subpopulations (≥ 20%) MAO‐B inhibitor use (Yes: *n* = 115, No: *n* = 201) Hoehn and Yahr stage (1: *n* = 78, 2: *n* = 238) RBDSQ (≥ 5: *n* = 85, < 5: *n* = 230) Data‐driven subphenotype (diffuse malignant: *n* = 59, nondiffuse malignant: *n* = 257)	At week 52:^b^ *n* = 96 (30.4%) • 32 in low‐dose (30.5%) • 33 in high‐dose (31.1%) • 31 in placebo (29.5%)	Primary: Efficacy^d^ Assessed through Change in MDS‐UPDRS Parts 1, 2 & 3 Secondary: NR	On vs. not on MAO‐B inhibitors Significant difference in MDS‐UPDRS Part 3 but not 1 and 2 Hoehn and Yahr stage 2 vs. stage 1 Significance difference in MDS‐UPDRS Part 3 but not 1 and 2 With vs. without REM sleep disorder No difference in MDS‐UPDRS Parts 1, 2 and 3 Diffuse vs. nondiffuse malignant phenotypes Significant difference in MDS‐UPDRS Part 3 but not 1 and 2 Exploratory subpopulations Significant differences were reported in subtypes with rapidly progressing disease vs. their nonrapidly progressing counterparts	NR

Brys et al., 2019 Cinpanemab	RCT ‐ Randomised ‐ Double‐blind ‐ Multicentre	16 weeks	PART 1 Total: *n* = 48 HVs Intervention (*n* = 34) 1 mg/kg (*n* = 3) 5 mg/kg (*n* = 7) 15 mg/kg (*n* = 6) 45 mg/kg (*n* = 6) 90 mg/kg (*n* = 6) 135 mg/kg (*n* = 6) Comparator: Placebo (*n* = 14) PART 2 Total: *n* = 18 PD patients Intervention (*n* = 12) 15 mg/kg (*n* = 6) 45 mg/kg (*n* = 6) Comparator: Placebo (*n* = 6)	At week 16: PART 1 *n* = 1 (2.94%) • 1 in 135 mg/kg (16.7%) PART 2 *n* = 0	Primary: Safety assessed through TRAEs, AEs, Serious Aes, Laboratory Tests, Vital Signs, ECG and physical and neurological examination Secondary: NR	PART 1 TRAEs: *n* = 6 (12.5%) 2 in placebo (14.3%), 4 in intervention (11.8%) By group: 1 in 15 mg/kg (16.7%), 1 in 90 mg/kg (16.7%), 2 in 135 mg/kg (33.3%) AEs: *n* = 26 (54.2%), Serious AEs: *n* = 1 (deemed related and in 135 mg/kg group), Deaths: *n* = 0 PART 2 TRAEs: *n* = 2 (11.1%) 1 in placebo (16.7%), 1 in intervention (8.3%) By group: 1 in 45 mg/kg (16.7%) Total AEs: *n* = 15 (83.3%), Serious AEs: *n* = 1 (deemed unrelated), Deaths: *n* = 0 BOTH PARTS laboratory tests, vital signs, examination, ECG: nothing clinically relevant	NR

Lang et al., 2022 Cinpanemab SPARK Trial^∗∗^	RCT ‐ Randomised ‐ Double‐blind ‐ Multicentre	52 weeks	Total: *n* = 357 PD Patients Intervention (*n* = 257) Doses: 250 mg (*n* = 55), 1250 mg (*n* = 102), 3500 mg (*n* = 100) Comparator: Placebo (*n* = 100)	At week 52: *n* = 167 (46.8%) • 26 of 250 mg (47.3%) • 55 of 1250 mg (53.9%) • 49 of 3500 mg (49%) • 47 of placebo (47%)	Primary: Efficacy assessed through change in MDS‐UPDRS combined score (Parts 1, 2 & 3) Secondary: Efficacy and safety Efficacy assessed through MDS‐UPDRS Parts 1, 2 and 3 separately and DaT‐Spect Safety assessed through AEs and Serious AEs	MDS‐UPDRS combined score: 250mg vs placebo mean difference (95% CI) −0.3 (−4.9–−4.3) 1250 mg vs placebo mean difference (95% CI) 0.5 (−3.3–4.3) 3500 mg vs placebo mean difference (95% CI) 0.1 (−3.8–4.0)	SAFETY AEs: *n* = 291 (81.1%) 80 in placebo (80.0%), 211 in intervention (82.1) Serious AEs were consistent across all groups but were not explicitly stated if they were due to the administration of placebo or intervention. EFFICACY All secondary efficacy‐related outcomes reported no significant differences.

Lang et al., 2022 Cinpanemab SPARK Trial^∗∗^	Open‐label extension of the SPARK trial ‐ Blinded to dose allocation ‐ Placebo group becomes delayed‐start cohort, with randomised doses in the same ratio and intervention becomes early‐ start cohort ‐ Multicentre	60 week extension (up to 112 weeks)	Total: *n* = 289 mITT PD Patients Early‐start cohort (*n* = 207) 250 mg (*n* = 41) 1250 mg (*n* = 89) 3500 mg (*n* =77) Comparator: Delayed‐start cohort (*n* = 82) Doses not separately reported	At week 44 of the extension: *n* = 73 (25.3%) • 13 in 250 mg (31.7%) • 27 in 1250 mg (30.3%) • 18 in 3500 mg (23.4%) • 15 in delayed‐start (18.3%)	Primary: Efficacy Assessed through change in MDS‐UPDRS combined score (Parts 1, 2 & 3) Secondary: Efficacy and Safety Efficacy assessed through MDS‐UPDRS Parts 1, 2 and 3 separately and DaT‐Spect Safety assessed through AEs and Serious Aes	MDS‐UPDRS combined score: 250 mg early‐ vs delayed‐start cohort mean difference (95% CI) −0.9 (−5.6–3.8) 1250 mg early‐ vs delayed‐start cohort mean difference (95% CI) 0.6 (−3.3–4.4) 3500 mg early‐ vs delayed‐start cohort difference (95% CI) −0.8 (−4.6–3.0)	SAFETY Safety was not reported separately for this phase, but pooled data from both periods suggest AEs were similar across groups. EFFICACY All secondary efficacy‐related outcomes reported no significant differences.

Hutchison et al., 2024 Cinpanemab SPARK Trial^∗∗^	Planned secondary imaging analysis of the SPARK trial ‐ Assessing secondary imaging outcomes from the original trial ‐ At week 52, the placebo group transitions to the delayed‐start cohort	Up to 112 weeks	Total: *n* = 357 PD Patients Intervention (*n* = 257) 250 mg (*n* = 55) 1250 mg (*n* = 102) 3500 mg (*n* = 100) Comparator: Placebo (*n* = 100)	At week 96: *n* = 147 (41.2%) • 25 in 250 mg (45.5%) • 44 in 1250 mg (43.1%) • 40 in 3500 mg (40.0%) • 38 in placebo/delayed‐start (38.0%)	Primary: Efficacy^e^ assessed with DaT‐SPECT and MRI Secondary: NR	DaT‐SPECT Striatal binding ratios decreased across all groups, mostly insignificantly. A significant difference was seen at Week 24 in the ipsilateral caudate (1250 mg and 3500 mg vs placebo), but not at Weeks 52 or 96. MRI In the 3500 mg group, substantia nigra pars compacta volume was significantly higher vs placebo at Week 24 only. Caudate volume was significantly higher in the 3500 mg group at Weeks 24 and 52, and in the 250 mg group at Week 24.	NR

Boström et al., 2024 Exidavnemab M19‐034	RCT ‐ Randomised ‐ Double‐blind ‐ Single‐centre	9 days	Total: *n* = 50HVs Intervention (*n* = 39) 100 mg (*n* = 6) 300 mg (*n* = 10) 1000 mg (*n* = 6) 3000 mg (*n* = 6) 6000 mg (*n* = 9) Comparator: Placebo (*n* = 11)	At day 9: *n* = 1 (2%) • 1 in placebo (9.09%)	Primary: Safety^f^ Assessed through TRAEs, AEs, severe AEs laboratory tests, vital signs and ECG Secondary: NR	BOTH TRIALS No data for AEs were provided for the placebo groups or for any specific doses in either trial. TRAEs in intervention groups *n* = 6 out of the 85 (7.1%) AEs in the intervention groups were reported: *n* = 49 out of the 85 (57.6%), severe AEs: *n* = 0, deaths: *n* = 0 M19‐034 ONLY Vital signs: 1 in 1000 mg group had 1 reading of 160 mmHg systolic pressure (45 above baseline) but second measurement taken showed normal. Nothing else was clinically relevant. Laboratory tests and ECG: nothing clinically relevant	NR

Boström et al., 2024 Exidavnemab M19‐465	Open‐label clinical trial ‐ Randomised ‐ Open‐label ‐ Single‐centre	9 days	Total: *n* = 48 HVs Intervention (*n* = 48) 300 mg (*n* = 8) 1000 mg (*n* = 8) 3000 mg (*n* = 24) 6000 mg (*n* = 8) Comparator: None	At day 9: *n* = 2 (4.17%) • 2 in 3000 mg (8.33%)	Primary: Safety Assessed through TRAEs, AEs, Laboratory Tests, Vital Signs and ECG Secondary: NR	AEs reported above with M19‐034. ECG: 1 in 100mg had asymptomatic ventricular ectopy on Day 1 but this resolved the same day. Nothing else was clinically relevant. Laboratory tests and vital signs: nothing clinically relevant	NR

Buur et al., 2024 Lu‐AF82422	RCT ‐ Randomised ‐ Double‐blind ‐ Single‐centre	12 weeks	COHORT A Total: *n* = 59 HVs Intervention (*n* = 41) 75 mg (*n* = 6) 225 mg (*n* =6) 750 mg (*n* = 6) 2250 mg (*n* = 9) 4500 mg (*n* = 8) 9000 mg (*n* = 7) Comparator: Placebo (*n* = 17) COHORT B Total: *n* = 15 PD Patients Intervention (*n* = 12) 2250 mg (*n* = 6) 9000 mg (*n* = 6) Comparator: Placebo (*n* = 3)	At week 12: COHORT A *n* = 2 (3.39%) • 1 in 2250 mg^c^ (12.5%) • 1 in placebo (5.88%) COHORT B *n* = 0	Primary: Safety Assessed through AEs, Laboratory Tests, Vital Signs, ECG, Weight, Blood Closure Time and C‐SSRS Secondary: NR	COHORT A AEs: *n* = 31 (53.4%) 9 in Placebo (52.9%), 22 in intervention (53.7%) By group: 4 in 75 mg (66.7%), 5 in 225 mg (83.3%), 1 in 750 mg (16.7%), 4 in 2250 mg (50.0%), 4 in 4500 mg (50%), 4 in 9000 mg (57.1%) Severe AEs: *n* = 0, Deaths: *n* = 0 COHORT B AEs: *n* = 10 (66.7%) 2 in placebo (66.7%), 8 in intervention (66.7%) By group: 3 in 2250 mg (50.0%), 5 in 9000 (83.3%) Severe AEs: *n* = 0, Deaths: *n* = 0 BOTH COHORTS laboratory tests, vital signs, ECG, weight, blood closure time and C‐SSRS: nothing clinically relevant	NR

Abbreviations: AEs, adverse events; CGl‐I, clinical global impression‐improvement; CI, confidence interval; C‐SSRS, Columbia‐Suicide Severity Rating Scale; DaT‐SPECT, dopamine transporter single photon emission computed tomography; ECG, electrocardiogram; HVs, healthy volunteers; kg, kilograms; LEDD, levodopa equivalent daily dose; MAO‐B, monoamine oxidase B; MDS‐UPDRS, Movement Disorder Society‐Unified Parkinson’s Disease Rating Scale; mg, milligrams; mITT, modified intention‐to‐treat; MoCA, Montreal Cognitive Assessment; MRI, magnetic resonance imaging; NR, not reported; PD, Parkinson’s disease; PGI‐C, patient global impressions of change; PPMI, Parkinson’s progression markers initiative; RBDSQ, Rapid Eye Movement Sleep Behaviour Disorder Screening Questionnaire; REM, rapid eye movement; SE‐ADL, Schwab and England activities of daily living; TRAEs, treatment‐related adverse events.

^∗^Participants were from the PASADENA RCT^14^ except the PPMI cohort.

^∗∗^Participants were from the Spark RCT^17^.

^a^Mean was calculated across the primary outcomes (MDS‐UPDRS Parts 2 and 3); reported with an approximation symbol (≈;) to 0 decimal places.

^b^Not reported; data assumed to concur with the original PASADENA RCT^14^.

^c^Participants withdrew before dosing.

^d^Outcomes reported briefly because this is an exploratory post hoc analysis.

^e^Outcomes reported as secondary in nature because these were prespecified as secondary outcomes of the original SPARK RCT^17^.

^f^AEs data from both trials were pooled together.

Table 2 reports demographic data. Ethnicities representing less than 20% of a study population were grouped as “Other”.

**TABLE 2:** Patient demographic.

Reference	Monoclonal antibody	Location (s)	Participants (*n* = number of participants)	Age (years)	Sex	Ethnicity	Hoehn and Yahr stage
Schenk et al., 2017	Prasinezumab^∗^	USA	*n* = 40 HVs	Median: 37 Range: 21–58	M: 37.5% F: 62.5%	White: 57.5% African American: 35% Other: 7.5%	N/A

Jankovic et al., 2018	Prasinezumab^∗^	USA	*n* = 80 PD Patients	Median: 58 Range: 43.78	M: 80% F: 20%	White: 97.5% Other: 2.5%	Stage 1: 12.5% Stage 2: 71.3% Stage 3: 16.3%

Pagano et al., 2022 (RCT)^∗^	Prasinezumab^∗^	Austria, France, Germany, Spain and USA	*n* = 316 PD Patients	Median: 61 Range: 40–80	M: 67.4% F: 32.6%	White: 83.2% Other: 16.8%	Stage 1: 24.7% Stage 2: 75.3%

Pagano et al., 2022 (open‐label extension)^∗^	Prasinezumab^∗^	Austria, France, Germany, Spain and USA	*n* = 309 PD Patients^a^	Median: ≈61 Range: ≈40–80	M: ≈67.4% F: ≈32.6%	White: ≈83.2% Other: ≈16.8%	Stage 1: ≈24.7% Stage 2: ≈75.3%
Pagano, Monnet et al., 2024^∗^	Prasinezumab^∗^	Austria, France, Germany, Spain and USA	*n* = 271 PD Patients *n* = 270 PPMI Patients^b^	PD Patients Mean: 59.9 SD: 9.0 PPMI Patients Mean: 61.2 SD: 9.3	PD Patients M: 69.4% F: 30.6% PPMI Patients M: 70.1% F: 29.9%	PD Patients^a^ White: ≈83.2% Other: ≈16.8% PPMI Patients^d^ White: ≈92.4% Other: ≈7.6%	PD Patients Stage 1: 25.8% Stage 2: 74.2% PPMI Patients^e^ Stage 1: ≈23.8% Stage 2: 76.2% Stage 3‐5: ≈0.00%

Pagano, Taylor et al., 2024^∗^	Prasinezumab^∗^	Austria, France, Germany, Spain and USA	*n* = 316 PD Patients^a^	Median: 61 Range: 40–80	M: 67.4% F: 32.6%	White: 83.2% Other: 16.8%	Stage 1: 24.7% Stage 2: 75.3%

Brys et al., 2019	Cinpanemab	USA	PART 1 *n* = 48 HVs PART 2 *n* = 18 PD Patients	PART 1 Median: 50 Range: 40–65 PART 2 Median: 64 Range: 47–75	PART 1 M: 60.4% F: 39.6% PART 2 M: 72.2% F: 27.8%	PART 1 White: 75% African American: 25% PART 2 White: 100%	PART 1 N/A PART 2 Stage 1: 16.7% Stage 2: 83.3%

Lang et al., 2022 (RCT)^∗∗^	Cinpanemab	Austria, Canada, France, Germany, Israel, Italy, Spain, UK, USA	*n* = 357 PD Patients	Mean: 60.1 SD: 9.0	M: 70.0% F: 30.0%	White: 91% Other: 9%	Stage 1: 24% Stage 1.5: 6% Stage 2: 65% Stage 2.5: 4% Stage 3: 1%

Lang et al., 2022 (Open‐label extension)^∗∗^	Cinpanemab	Austria, Canada, France, Germany, Israel, Italy, Spain, UK, USA	*n* = 289 mITT PD Patients^c^	Mean: ≈60.1 SD: ≈9.0	M: ≈70.0% F: ≈30.0%	White: ≈ 91% Other: ≈ 9%	Stage 1: ≈24% Stage 1.5: ≈6% Stage 2: ≈65% Stage 2.5: ≈4% Stage 3: ≈1%

Hutchison et al., 2024^∗∗^	Cinpanemab	Austria, Canada, France, Germany, Israel, Italy, Spain, UK, USA	*n* = 357 PD patients	Mean: 60.1 SD: 9.0	M: 70.0% F: 30.0%	White: 91% Other: 8%	Stage 1: 24% Stage 1.5: 6% Stage 2: 65% Stage 2.5: 4% Stage 3: 1%

Boström et al., 2024 (M19‐034)	Exidavnemab	USA	*n* = 50 HVs	Mean: 46.5 SD: 10.7	M: 78.0% F: 22.0%	White: 68% African American: 24% Other: 8%	N/A

Boström et al., 2024 (M19‐465)	Exidavnemab	USA	*n* = 48 HVs	Mean: 41.2 SD: 9.4	M: 75.0% F: 25.0%	Asian: 100%	N/A

Buur et al., 2024	Lu‐AF82422	USA	COHORT A *n* = 59 HVs COHORT B *n* = 15 PD patients	COHORT A Mean: 38 SD: 9.1 COHORT B Mean: 63 SD: 7.7	COHORT A M: 60.3% F: 39.7% COHORT B M: 46.7% F: 53.3%	COHORT A Asian: 36.2% White: 25.9% African American: 24.1% COHORT B White: 80.0% Other: 20.0%	COHORT A N/A COHORT B^f^ Stage 1–3: 100%

*Note:* An approximation symbol (≈;) is used when statistics were not directly reported but were inferred from the original study cohort. F, female; HVs, healthy volunteers; M, male; mITT, modified intention‐to‐treat.

Abbreviations: N/A, not applicable; PD, Parkinson’s disease; PPMI, Parkinson’s progression markers initiative; SD, standard deviation; UK, United Kingdom; USA, United States of America.

^∗^Participants were all from the PASADENA RCT^14^ except the PPMI cohort.

^∗∗^Participants were all from the SPARK RCT^17^.

^a^Not reported; data assumed to concur with the original PASADENA RCT^14^.

^b^Postpropensity‐weighted statistics for the PPMI cohort were not always reported.

^c^Not reported; data assumed to concur with the original SPARK RCT^17^.

^d^Postpropensity weighting not reported; data assumed to concur with the original PPMI study.

^e^Postpropensity weighting not reported. Original study reported 2 participants (0%) with scores ≥ 3 meaning almost all were at Stages 1‐2; percentage of Stage 1 was inferred.

^f^Stage‐specific data not reported.

### 2.4. Quality Assessment

**FIGURE 4:**
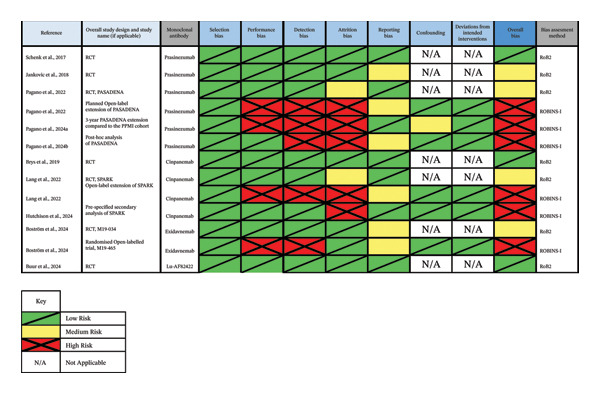
Risk of bias assessment for included studies. Abbreviations: N/A, not applicable; RCT, randomised controlled trial; RoB2, cochrane Risk of Bias 2; ROBINS‐I, risk of bias in nonrandomised studies of intervention.

Randomised controlled trials (RCTs) were assessed using the Cochrane Risk of Bias 2 (RoB2) tool [12]. Nonrandomised studies were evaluated using the Risk of Bias in Nonrandomised Studies‐of Interventions (ROBINS‐I) tool [13]. Assessment of risk of bias was conducted independently by three reviewers; results are reported in Figure 4. Sensitivity analysis was performed to exclude high‐risk studies.

## 3. Results

### 3.1. Study Selection

One thousand five hundred and nine unique articles were identified—narrowing to 10 for analysis after screening. The PRISMA flow diagram (Figure 3) outlines the selection process [11].

### 3.2. Study Findings

The results with available confidence intervals are shown in Table 1. Efficacy was assessed for Prasinezumab and Cinpanemab. Efficacy for Exidavnemab and Lu‐AF82422 has not been evaluated yet.

### 3.3. Prasinezumab: Efficacy

The PASADENA RCT failed to meet its primary outcome with no significant changes in the sum of scores on Parts I, II and III of the MDS‐UPDRS [14]. The PPMI vs. PASADENA comparison focused on MDS‐UPDRS Parts 2 and 3. Propensity‐weighted analysis against the PPMI cohort showed lower mean scores across both parts [15]. Reported differences, with 80% confidence intervals, ranged from −1.82 (−2.44–−1.20) for MDS‐UPDRS Part 2 to −7.26 (−8.59–−5.93) for MDS‐UPDRS Part 3. Both were comparing the early‐start to the PPMI cohort. MDS‐UPDRS Part 1 and Levodopa Equivalent Dose Calculator were secondary outcomes which also achieved significant results. The post hoc analysis of the PASADENA RCT found potential reductions in Part 3 among rapidly progressing PD subgroups [16].

### 3.4. Cinpanemab: Efficacy

Cinpanemab showed no significant effect over placebo for any primary outcomes [17]. Secondary analysis suggested early imaging differences—reduced dopamine transporter loss on DaT‐SPECT and less nigrostriatal atrophy on MRI—but these were not sustained in the final analysis [18].

### 3.5. Safety

Some of the included studies reported AEs rather than TRAEs, with similar rates across all groups. TRAEs ranged from 7.0% to 38.7%, and some patients experienced serious AEs, e.g., 4.8%, 6.7% and 7.5% in the placebo, low‐dose and high‐dose cohort, respectively, in the 2022 PASEDENA RCT [14]. However, this percentage dropped below 2% in the low‐ and high‐dose cohorts when treatment relation was determined. Furthermore, these treatment‐related serious AEs did not lead to discontinuation of treatment. This pattern of serious AEs was replicated across other trials with even lower percentages. One suicide occurred in Prasinezumab’s 52‐week extension, but it was deemed unrelated to the trial [14]. Other safety measures, including vital signs and clinical examinations, were reported inconsistently; when available, the results were generally within the normal range, with occasional outliers deemed clinically insignificant.

### 3.6. Risk of Bias Findings

Risk of bias varied across studies: three were low, four were medium and six were high, as shown in Figure 4.

### 3.7. Sensitivity Analysis

Six high‐risk‐of‐bias studies were identified. When these were excluded, Cinpanemab’s efficacy was unchanged. However, excluding these high‐risk‐of‐bias studies resulted in the removal of the PPMI vs. PASADENA comparison and the post hoc analysis of the PASADENA trial. Taken together, these exclusions attenuated the significance of Prasinezumab’s efficacy. Safety conclusions were largely unchanged.

## 4. Discussion

### 4.1. Summary of Results

Ten articles were identified, some covering multiple studies. Prasinezumab was assessed in six studies, Cinpanemab in four, Exidavnemab in two, and Lu‐AF82422 in one. Efficacy was evaluated only for Prasinezumab and Cinpanemab, with mixed results: Prasinezumab showed some efficacy, whilst Cinpanemab demonstrated minimal efficacy. Most subjects tolerated mAbs well. The safety of Exidavnemab and Lu‐AF82422 supports future clinical efficacy assessments.

### 4.2. Prasinezumab: Efficacy

Prasinezumab findings were variable. In the RCT by Pagano et al., primary and most secondary outcomes were not met [14]. Nominally significant differences were reported in MDS‐UPDRS Part 3 for the low‐dose cohort and in Montreal Cognitive Assessment scores for the high‐dose cohort, but both used 80% confidence intervals without adjustment for multiplicity, limiting reliability. The improvement in the low‐ but not high‐dose group was biologically unexpected. Whilst U‐shaped dose‐responses do exist, due to the earlier mentioned limitations, these results are more plausibly a statistical anomaly. [19].

The 52‐week PASADENA open‐label extension found no significant differences across outcomes, although the lack of a placebo group limits interpretation. [14] Comparisons between intervention cohorts could have obscured actual treatment effects so negative results should be interpreted cautiously.

Some evidence of Prasinezumab efficiency was shown in the PASADENA vs. PPMI analysis, which reported improvements in MDS‐UPDRS Parts 2 and 3 across all dosing groups with some secondary outcomes also reaching statistical significance [15]. This occurred at Year 1 of the extension, with greater differences in the subsequent 2 years. This may indicate that treatment effects require time to emerge, or that the insidious nature of PD means 1 year is insufficient to significant changes [20]. To compare, Lecanemab, a different mAb studied in Alzheimer’s disease (AD), showed benefits at 6 months, increasing until the 18‐month final endpoint, supporting the plausibility of delayed efficacy [15].

Whilst the Prasinezumab study by Pagano et al. demonstrated some positive results in MDS‐UPDRS Parts two and three across multiple cohorts, it relied on 80% confidence intervals a nonplacebo comparator and an open‐label design [15]. These are subject to residual confounding, and potential performance and detection biases. Whilst the number of positive results makes bias alone less likely, more robust studies are needed to confirm these findings.

Whilst the original 52‐week PASADENA trial showed no significant changes, a post hoc analysis of study participants demonstrating rapidly progressing PD subtypes revealed a slower decline in MDS‐UPDRS Part 3 compared to the placebo group [16]. Several plausible reasons might explain such a finding. Firstly, participants with rapidly progressive PD would have amassed a greater disease burden over the trial duration than nonrapidly progressive participants, improving the likelihood of such changes being detected and effectively giving a greater signal‐to‐noise ratio [15]. Secondly, one of these subgroups used monoamine oxidase‐B (MAO‐B) inhibitors as a proxy for rapidly progressive PD [16]. As the decisions to use MAO‐B inhibitors were made by the treating physicians rather than as part of a trial protocol, this apparent subgroup of rapidly progressive PD may have incorporated additional variables and so may not have been a discretely defined group [16]. Lastly, rapid disease progression could be explained by more widespread alpha‐synuclein accumulation providing more targets for Prasinezumab to act upon. Future development of in vivo pathological alpha‐synuclein biomarkers may allow such hypotheses to be tested [21]. However, the post hoc nature of the analysis was itself a significant limitation to the results, and as such the apparent improvement in the rapidly progressive PD cohort might have had other explanations.

Evidence from the 3‐year open‐label extension and post hoc analyses suggests that Prasinezumab may require extended time to demonstrate benefit, reflecting the slow progression of PD. These findings raise the possibility that intervention after symptom onset may be too late, and that a potential prophylactic approach could prove more effective.

The positive results should be interpreted with caution. The significant improvements in MDS‐UPDRS Part 3 in rapidly progressing PD subtypes were limited by cohort overlap, repeated analyses of the same population and lack of independent replication [16]. Open‐label extensions and post hoc designs further increase bias risk (Figure 4). Sensitivity analyses removed most positive findings, leaving only the minimal effects in the PASADENA RCT [14]. Therefore, Prasinezumab’s potential benefits remain uncertain and require confirmation in future studies.

### 4.3. Cinpanemab: Efficacy

Cinpanemab showed no significant clinical efficacy in the SPARK RCT, failing to meet any primary or secondary endpoints, including MDS‐UPDRS scores and imaging outcomes [17]. A secondary analysis reported early reductions in caudate and substantia nigra volume loss in higher‐dose groups, suggesting a temporary protective effect, but these findings were not sustained at later endpoints [18]. This transient efficacy may indicate a slight effect that ultimately is outweighed by PD progression.

### 4.4. Prasinezumab: Safety

Prasinezumab demonstrated a generally favourable safety profile across four clinical trials, with TRAEs showing only minor differences from placebo. In short‐term trials, TRAE rates differed between treatment and placebo arms by no more than 3.3% [22, 23]. Longer trials showed larger differences in TRAEs, with a 12% increase in the PASADENA RCT in the high‐dose cohort compared to placebo, though the significance is unclear [14]. This rise in TRAEs with prolonged mAb use is not unexpected and may relate to immunogenicity or off‐target effects [24]. Some serious AEs were reported in the PASADENA trial (both across the RCT and open‐label portion), reaching 7.5% of the total high‐dose cohort during the RCT [14]. However, when treatment relation was determined, the frequency of serious AEs reported was less than 2% of the cohort. Moreover, no patients in any of the identified studies discontinued treatment because of a serious AE. The suicide reported in the PASADENA open‐label extension was deemed unrelated to treatment [14]. Nonetheless, reliance on investigator‐led attribution and open‐label designs introduces detection bias, which may affect reported rates and the appointment of treatment relation.

### 4.5. Cinpanemab: Safety

Cinpanemab demonstrated a favourable safety profile. TRAEs were low in PD patients in the study by the authors in [21]. In the SPARK RCT, the 2% difference between placebo and intervention groups suggests that AEs were largely unrelated to treatment [17]. A limitation of the SPARK safety data is the pooling with its open‐label extension, which may obscure AEs from prolonged, unblinded use, raising potential reporting bias. Nonetheless, these safety concerns are of limited clinical relevance, as Cinpanemab was discontinued due to lack of efficacy rather than safety issues.

### 4.6. Exidavnemab: Safety

Exidavnemab demonstrated a favourable safety profile, with six TRAEs reported among 85 HVs, and none were deemed serious [25]. However, the strength of this conclusion is limited by the study design. Placebo data were not reported, preventing accurate comparison, and results from M19‐034 and the noncomparator M19‐465 were pooled, further obscuring the true safety profile. As this study involved HVs, tolerability should ideally be confirmed in PD patients before larger trials can proceed.

### 4.7. Lu‐AF82422: Safety

Lu‐AF82422 showed similar AE rates between placebo and intervention, with a 1.2% difference and no serious AEs in HVs [26]. In PD patients, 66.7% of both placebo and intervention cohorts experienced AEs. Although treatment relation was not assessed, the similarity between groups suggests a favourable safety profile. This aligns with preliminary data in multiple system atrophy, where a conference abstract reported good tolerability and trends towards efficacy, though not statistically significant [27]. As this was not peer‐reviewed, the findings provide context rather than firm conclusions.

### 4.8. Epitope Targeting as a Potential Explanation for Trial Outcomes

Prasinezumab and Cinpanemab bind to the C‐terminus and N‐terminus of alpha‐synuclein, respectively [28]. According to Liu et al., reduced efficacy of N‐terminal targeting was not attributable to differences in binding affinity but rather to diminished functional impact on alpha‐synuclein aggregation [29]. Their hypothesis proposes that the C‐terminal region, enriched in negatively charged residues, may play a key role in pathological interactions with cellular membranes and other alpha‐synuclein aggregates, thereby contributing to disease propagation [29]. Consequently, C‐terminal‐directed binding might theoretically produce therapeutic effects by sterically inhibiting aggregate cell interactions, thereby reducing cell‐to‐cell transmission, neuroinflammatory activation and autophagy dysfunction [30]. The post hoc analysis finding of a slower decline in MDS‐UPDRS Part 3 for rapidly progressive PD treated with Prasinezumab [16]. In contrast to the lack of clinical improvement with Cinpanemab, the authors in [17] may provide some support for that hypothesis. Further evidence from ongoing and future clinical trials involving Prasinezumab may provide additional support for the therapeutic relevance of C‐terminal targeting. Lu‐AF82422 and Exidavnemab also bind preferentially to the C‐terminus of alpha‐synuclein, but efficacy data are currently lacking for these agents [29, 31].

### 4.9. Comparison With the Previous Systematic Review

The previous systematic review covering mAbs in PD was conducted by Rodger et al. though they also included other alpha‐synuclein–targeting modalities such as vaccines [32]. They discussed Prasinezumab and Cinpanemab but did so with only one peer‐reviewed study [23]. Although the PASADENA trial was cited [32], no results were presented in tables, and most data were only briefly discussed. Prasinezumab and Cinpanemab were deemed ineffective in that review. Our current systematic review aligns with the conclusions by Rodger et al. regarding Cinpanemab, but in contrast, highlights some positive outcomes for Prasinezumab. This difference in results reflects both the more up to date time frame of our current study and the in‐depth data extraction and analysis used.

The efficacy of mAbs‐targeting alpha synuclein in PD remains uncertain, as suggestive outcomes were either not sustained at later endpoints or were limited by high risk of bias and methodological issues. Future studies are required to further clarify this field, especially regarding the possible investigation of such mAbs in presymptomatic PD. Alpha‐synuclein–targeted mAbs in PD do demonstrate a favourable safety profile across studies, supporting the current consensus of mAb safety [10]. However, some rare serious AEs do occur, and these should be monitored for carefully in future trials. Compared with Rodger et al.’s review, the additional detailed data presented here strengthen the evidence base for the overall safety of mAbs‐targeting alpha synuclein in PD and provide some hope for potential future evidence of efficacy.

### 4.10. Future Direction

MAbs are not currently approved for clinical use in PD. Prasinezumab’s possible disease‐modifying potential requires verification from more robust RCTs. An ongoing RCT assessing Prasinezumab’s potential in 586 PD patients, primary effects on motor progression, is being conducted by Nikolcheva et al. [33]. As an RCT, it has a more robust methodology than prior open‐label studies and post hoc analyses. The results could be important for evaluating Prasinezumab’s disease‐modifying potential on motor symptoms and the broader possibilities of mAbs in PD. If the results are positive, investigating DMTs targeted at nonmotor symptoms would still represent another potential area of research. If negative, they could provide clarity and help redirect resources towards alternative treatments such as vaccines. The potential to test Prasinezumab prophylactically could remain even if results from this trial turn out to be insignificant, especially if the results confirm positive findings with aggressive subtypes.

The AHEAD 3‐45 study entails the administration of prophylactic Lecanemab to asymptomatic individuals with elevated amyloid biomarkers suggestive of AD [34]. In PD, prodromal features such as hyposmia and urinary incontinence have been proposed to help identify PD earlier, and other biomarkers are under investigation [35, 36]. Identifying such markers could allow targeted prophylactic testing of Prasinezumab and allow an approach analogous to the AHEAD 3‐45 study to occur.

Cinpanemab has been discontinued, and this present study results are in line with that decision despite some minor efficacy suggested in imaging [18]. In contrast, Exidavnemab and Lu‐AF82422 should progress to longer‐term trials in PD patients to assess efficacy and safety [25, 26]. The EXIST trial, an ongoing RCT, has begun assessing Exidavnemab’s tolerability in PD patients, though no timeframe or peer‐reviewed data are currently available [37].

MEDI1341 is another alpha‐synuclein–targeting mAb but was excluded from our study results as only a conference abstract was available at the time of screening [38]. This abstract suggested some good tolerability of MEDI1341in HVs [38]. If confirmed in a peer‐reviewed article, these findings could justify testing this mAb in PD patients.

Combination mAb therapy could be an alternative approach, given the suggested tolerability of mAbs. Oncological studies have shown promising synergistic results with mAb combinations in cancer treatment [39]. The majority of the studies in this review utilised mAbs with different pathways. In contrast, two different mAbs (Trastuzumab and Pertuzumab) targeting the same human epidermal growth factor Receptor 2 suggested improved results when used together rather than individually in breast cancer studies [39]. Should trials of vaccines targeting α‐synuclein, such as UB‐312 for PD, prove successful, a speculative possibility would be combining them with prophylactic monoclonal antibodies as an alternative combination therapeutic.

Inokuchi and Shimamoto reported a reduction in PD incidence among patients treated with Romosozumab, a mAb for osteoporosis [40]. It showed a statistically significant reduction in PD compared to patients treated with parathyroid hormone receptor treatment [40]. Romosozumab was excluded from this study as it targets sclerostin in the Wnt/β‐catenin pathway, not alpha‐synuclein [40]. However, the findings suggest the potential of Wnt pathway modulation as an alternative treatment or preventative mechanism for PD.

### 4.11. Limitations of This Study

Considerable heterogeneity was present among the included studies, with different mAbs evaluated across varied populations and study designs, excluding a meta‐analysis. Therefore, positive trends should be interpreted cautiously.

The demographic analysis revealed that most subjects were white. Whilst some evidence suggests that PD may be more prevalent in White populations, genetic variations could limit the applicability of the positive results to other demographics [41]. Furthermore, this demographic finding from our included studies might represent underrepresentation rather than a genuine genetic difference. The overlapping cohorts also likely reduced the generalisability of the results as it lowered the total number of individual participants.

## 5. Conclusion

Our study has highlighted some potential for the use of alpha‐synuclein mAbs as DMTs in PD, although future research is required to help clarify this. Across our study and the broader literature, mAbs have demonstrated generally favourable safety profiles with some serious AEs that should be monitored for in future trials. The tantalising possibility of future studies focussing on alpha‐synuclein mAbs in presymptomatic PD remains. For now, the efficacy of these mAbs in PD remains uncertain and will require confirmation through additional well‐designed RCTs.

## Author Contributions

Daniel Coles contributed to the conception, organisation and execution of the research project; the design, execution and review and critique of the statistical analysis and the writing of the first draft and review and critique of the manuscript.

Anish Kalyana contributed to the execution of the research project and the review and critique of the manuscript.

Sameer Khalil contributed to the execution of the research project and the review and critique of the manuscript.

Dhyana Chauhan contributed to the execution of the research project and the review and critique of the manuscript.

Thiara Rupasinghe contributed to the execution of the research project; the execution and review and critique of the statistical analysis and the review and critique of the manuscript.

Charlie Costello contributed to the execution and review and critique of the statistical analysis and the review and critique of the manuscript.

Amit Batla contributed to the writing of the first draft and the review and critique of the manuscript.

Tim Young contributed to the conception and organisation of the research project and to the writing of the first draft and review and critique of the manuscript.

## Funding

No funding was received for this manuscript.

## Ethics Statement

The authors confirm that the approval of an institutional review board was not required for this work. Informed patient consent was not necessary for this work. The authors confirm that they have read the journal’s position on issues involved in ethical publication and affirm that this work is consistent with those guidelines.

## Conflicts of Interest

The authors declare no conflicts of interest.

## Data Availability

Data sharing is not applicable to this article as no datasets were generated or analysed during the current study.
